# Characterization of the Newly Isolated Lytic Bacteriophages KTN6 and KT28 and Their Efficacy against *Pseudomonas aeruginosa* Biofilm

**DOI:** 10.1371/journal.pone.0127603

**Published:** 2015-05-21

**Authors:** Katarzyna Danis-Wlodarczyk, Tomasz Olszak, Michal Arabski, Slawomir Wasik, Grazyna Majkowska-Skrobek, Daria Augustyniak, Grzegorz Gula, Yves Briers, Ho Bin Jang, Dieter Vandenheuvel, Katarzyna Anna Duda, Rob Lavigne, Zuzanna Drulis-Kawa

**Affiliations:** 1 Department of Pathogen Biology and Immunology, Institute of Genetics and Microbiology, University of Wroclaw, Wroclaw, Poland; 2 Division of Gene Technology, Catholic University of Leuven, Leuven, Belgium; 3 Department of Microbiology, Institute of Biology, The Jan Kochanowski University in Kielce, Kielce, Poland; 4 Department of Molecular Physics, Institute of Physics, The Jan Kochanowski University in Kielce, Kielce, Poland; 5 Division of Structural Biochemistry, Research Center Borstel, Leibniz-Center for Medicine and Biosciences, Borstel, Germany; UC Berkeley, UNITED STATES

## Abstract

We here describe two novel lytic phages, KT28 and KTN6, infecting *Pseudomonas aeruginosa*, isolated from a sewage sample from an irrigated field near Wroclaw, in Poland. Both viruses show characteristic features of *Pbunalikevirus* genus within the *Myoviridae* family with respect to shape and size of head/tail, as well as LPS host receptor recognition. Genome analysis confirmed the similarity to other PB1-related phages, ranging between 48 and 96%. *Pseudomonas* phage KT28 has a genome size of 66,381 bp and KTN6 of 65,994 bp. The latent period, burst size, stability and host range was determined for both viruses under standard laboratory conditions. Biofilm eradication efficacy was tested on peg-lid plate assay and PET membrane surface. Significant reduction of colony forming units was observed (70-90%) in 24 h to 72 h old *Pseudomonas aeruginosa* PAO1 biofilm cultures for both phages. Furthermore, a pyocyanin and pyoverdin reduction tests reveal that tested phages lowers the amount of both secreted dyes in 48-72 h old biofilms. Diffusion and goniometry experiments revealed the increase of diffusion rate through the biofilm matrix after phage application. These characteristics indicate these phages could be used to prevent *Pseudomonas aeruginosa* infections and biofilm formation. It was also shown, that PB1-related phage treatment of biofilm caused the emergence of stable phage-resistant mutants growing as small colony variants.

## Introduction

The aerobic gram-negative bacterium *Pseudomonas aeruginosa* is an opportunistic human pathogen, that causes chronic and acute infections of burn wounds, respiratory and urinary tracts [1]. Particularly vulnerable are patients with cancer, HIV infection and the immunocompromised or mechanically ventilated [2]. *P*. *aeruginosa* is also the major cause of morbidity and mortality in individuals suffering from cystic fibrosis [3]. This bacterium possesses a wide array of virulence factors that do not only cause extensive tissue damage, but also directly interfere with the human immune system [4]. Highly diverse species of *P*. *aeruginosa* with the capacity to form biofilms are ubiquitons in the environment and are naturally resistant to many drugs [5]. *P*. *aeruginosa* reversibly regulates gene expression and changes its phenotype and genotype in response to the environmental signals during the infection [6]. This provides a protective mode that allows microorganisms to survive in hostile environments and disperse seeding cells to colonize new niches with desirable conditions [7].

Due the recent increase in multidrug resistance of *P*. *aeruginosa* strains, the discovery of alternative prevention and treatment strategies is necessary. An interesting possibility is the use of bacteriophages (phages), viruses that infect and replicate within a bacterium [8]. The major advantages over antibiotics are the specificity of phages, their rapid isolation, and the ability to reach bacteria surrounded within a biofilm matrix [5,9]. Phage cocktails were commonly applied as alternative or as supportive treatments simultaneously with antibiotics, particularly in Georgia and Eastern Europe. Positive results were routinely obtained with the eradication of *Escherichia*, *Pseudomonas*, *Proteus*, *Klebsiella* and *Staphylococcus* clinical strains from various kinds of purulent infections [10–12].

Well characterized and widespread in nature is a group of Pb1-like phages within to the *Myoviridae* family. Their genomes are highly conserved (more than 85% of nucleotide identity) and organized in at least seven transcriptional blocks. The size varies between 64,427 and 66,530 bp with 88 to 95 encoded proteins [13]. Phages from this group do not encode a recognizable integrase, suggesting their obligatory virulent nature [14]. They have a solid, acid-resistant isometric capsid, with a diameter of ~74 nm and a contractile tail of ~140 nm [13]. They recognize lipopolysaccharide (LPS) as their host receptor [15].

Over the years, no less than 43 phages have been reported to be Pb1-like, mainly based on cross-DNA hybridization and morphological studies [16]. This virus group is described as suitable for application in phage therapy experiments, especially in phage cocktails [17]. Here we describe two new broad host range *Pbunalikevirus* phages KT28 and KTN6, which can be considered as therapeutic agents in the future. Their genomes have been sequenced and a detailed comparison with other members of the Pb1-like viruses was made. The morphology, burst size, and host range were established as well as viral particle stability, and LPS interactions. Moreover, different methods for biofilm eradication efficacy were investigated to evaluate phage potency in affecting biofilm forming bacterial cells.

## Materials and Methods

We confirm that the field studies did not involve endangered or protected species. No specific permits were required for water (sewage) sampling from the described field studies according to Polish Water Act (Dz.U. 2001 Nr 115 poz.1229. Prawo wodne).

### Isolation, propagation and purification of phages

The *Pseudomonas aeruginosa* PAO1 (ATCC 15692) strain purchased from the American Type Culture Collection was used as a host for phage propagation. Environmental water samples from irrigated fields in Wroclaw, Poland were centrifuged (15,000 g for 15 min) and the supernatant was filtered through a 0.22 μm Millex-GP filter (Merck Millipore, Germany) to remove bacterial debris. For phage propagation 0.5 ml of filtered water sample and 5 μl bacterial host strain, grown overnight in Mueller Hinton Broth (MHB) (Bio-Rad Laboratories, Hercules, CA, USA) were added to 5 ml of MHB and incubated at 37°C with agitation for 18h. Next, 10 ml of chloroform was added into the flask for an additional 10 min at 37°C. The bacterial debris was then removed by centrifugation at 5,000 × g for 15 min at 4°C and filtered through a 0.22 μm Millex-GP filter. For phage purification 25% polyethylene glycol 8000 (Acros organics, Geel, Belgium) was added and stirred overnight at 4°C and recovered by centrifugation at 4,600 × g for 45 min at 4°C. The pellet was suspended in 2 ml of phage buffer (10 mM Tris-HCl, 10 mM MgSO_4_, 150 mM NaCl, pH 7.5). Phage lysate was purified with CsCl-gradient ultracentrifugation as described by Ceyssens *et al*. (2008) [18] and dialyzed 3 times for 30 min against 250 volumes of phage buffer using Slide-A-Lyzer Dialysis Cassettes G2 (Thermo Fisher Scientific Inc, MA, USA). The phage titre of the solution was assessed using the double-agar layer technique as according to methods described previously [19].

### Electron microscopy

A filtered high-titer phage lysate was centrifuged at 25,000 g for 60 min. The pellet was washed twice in ammonium acetate (0.1 M, pH 7.0). Phages were deposited on copper grids with carbon-coated Formvar films and stained for 10 s with uranyl acetate (2%, pH 4.5) or phosphotungstate (2%, pH 7). Excess liquid was blotted off and phages were examined using a Zeiss EM 900 electron microscope in Laboratory of Microscopy Techniques, University of Wroclaw, Poland. The magnification was calibrated using T4 phage tail length (114 nm) as a standard.

### Burst size experiments

A one-step growth curve was performed according to the method of Pajunen *et al*. (2000) [20], with modifications. An equal volume of bacterial culture (at optical density at 600 nm of 0.4) was mixed with phage suspension (10^6^ pfu/ml) to obtain a multiplicity of infection of 0.01. Phages were allowed to adsorb for 8 min at 37°C, after which the mixture was diluted to 10^–4^. Triplicate samples were taken during 1 h at 5 min intervals and titrated.

### Sensitivity of phage particles to heat, chloroform and pH

The sensitivity to heat was determined by incubating phage suspension (10^8^ pfu/ml) in phage buffer at various temperatures (40, 50, 60, 70, and 80°C) for 5, 15, 30, 45 and 60 min. Chloroform sensitivity was determined by the incubation of equal volumes of phage suspension (10^8^ pfu/ml) and chloroform for 1 and 24 h at 4°C with intermittent shaking. The pH stability was tested by incubation of 100 μl of phage suspension (10^8^ pfu/ml) in 900 μl of universal buffer at pH range 2–12 (150 mM KCl, Janssen Chimica, Geel, Belgium; 10mM KH_2_PO_4_, VWR International, Leuven, Belgium; 10mM sodium citrate, Acros Organics; 10mM H_3_BO_3_, Acros Organics; adjusted to pH 1 to 13 with NaOH, or HCl). Phage titer was assessed after 1h incubation at room temperature. After incubation at the different conditions, the phage titer was assessed as previously described.

### Phage typing and phage receptor analysis

The lytic activity of isolated viruses was examined on 58 clinical *P*. *aeruginosa* strains from Military Hospital Nederoverheembeek, Brussels, Belgium [21] and compared to the activity of other Pb1-like phages: LMA2 and LBL3 (S1 Table). The phage specificity to particular bacterial receptors was tested on PAO1 mutants deficient in biosynthesis of A-band and B-band O-antigen, flagella, type IV pili, or alginate production (Table 1). For all phage experiments 4–6 h old bacterial cultures were used, unless otherwise stated. To determine bacterial susceptibility to phage-mediated lysis, a drop of the phage suspension (10^8^ pfu/ml) was put on a bacterial lawn and incubated at 37°C. The plates were checked after 4–6 h and again after 18 h for the presence of a lysis zone [19,22].

**Table 1:** Phage receptor identification on *P*. *aeruginosa* PAO1 mutants.

Bacterial strain	Phenotype	Origin	KT28	KTN6
**PAO1 (ATCC 15692)**	Wild type	American Type Culture Collection	+	+
**PAO1 Pirnay**	Wild type with inactive type IV pili	Military Hospital Nederoverheembeek, Brussels, Belgium, Dr. Jean-Paul Pirnay	+	+
**PAO1 Krylov**	Wild type	Military Hospital Nederoverheembeek, Brussels, Belgium, Dr. Jean-Paul Pirnay	+	+
**PAO1 Δrmd (A-, B+)**	Deficiency in D-rhamnose biosynthesis; lack of A-band LPS	Laboratory of Foodborne Zoonoses, Guelph, Canada, Andrew M. Kropinski	+	+
**PAO1 ΔrmLC (A-, B-, core-)**	Deficiency in L-rhamnose biosynthesis; truncate core region, lack of A-band and B-band LPS	Laboratory of Foodborne Zoonoses, Guelph, Canada, Andrew M. Kropinski	-	-
**PAO1 ΔwaaL (A-, B-)**	Lack of WaaL ligating O-polymer to core-lipid A; LPS is devoid of A-band and B-band, semirough (SR-LPS, or core-plus-one O-antigen)	Laboratory of Foodborne Zoonoses, Guelph, Canada, Andrew M. Kropinski	+	+
**PAO1 ΔwbpL (A-, B-)**	Lack of glucosyltransferase WbpL essential for initiation of both A-band and B-band synthesis	Laboratory of Foodborne Zoonoses, Guelph, Canada, Andrew M. Kropinski	-	-
**PAO1 ΔfliC ΔalgC ΔpilA**	Lack of flagella; lack of AlgC required for A-band, core oligosaccharide, and alginate biosynthesis; lack of type IV pili	Technical University Hamburg, Germany, Max Schöbert	-	-
**PAO1 ΔfliC wt algC ΔpilA**	Lack of flagella; lack of type IV pili	Technical University Hamburg, Germany, Max Schöbert	+	+
**PAO1 ΔfliC wt algC wt pilA**	Lack of flagella	Technical University Hamburg, Germany, Max Schöbert	+	+
**PAO1 wt fliC wt algC wt pilA**	Wild type	Technical University Hamburg, Germany, Max Schöbert	+	+

### LPS isolation

The isolation of LPS was performed with hot phenol/water according to Westphal *et al*. [23] with some modifications. Delipidated dried bacterial cells were re-suspended in 90% phenol and stirred overnight. Subsequently the hot phenol/water extraction protocol was applied, and both water and phenolic phases were collected, dialyzed and freeze-dried. Such obtained samples were used for further experiments.

### Phage inactivation by LPS

The phage inactivation by LPS was performed as previously described by Kropinski [24], with some modifications. The LPS was suspended in sterile milliQ water to a concentration ranging from 400 μg/ml to 0.78 μg/ml. Phage particles at final concentration of 3 × 10^3^ pfu/ml were added to each dilution of LPS. The suspensions were incubated at 37°C for 1 h on an orbital shaker (180 rpm). After incubation, the phage titer was assessed by the double agar layer technique. The results were expressed as phage infectivity inhibition calculated as LPS concentration that caused 50% inhibition of phage infectivity.

### LPS-binding assay

The LPS-binding assay basing on biotinylated phages was developed in our lab. LPS was diluted in carbonate-bicarbonate buffer (pH 9.6) to a concentration of 10 μg/ml and 100 μl was added to 96-well flat-bottom microtitre plate (MaxiSorp, NUNC), incubated 4 h at 37°C and then dried overnight at room temperature (RT). CsCl ultracentrifugation purified phage particles (~10^8^ pfu/ml) were biotinylated with EZ-linked sulfo-NHS-biotin (Pierce, Rockford, USA) at final concentration 100 μg/ml in cold carbonate-bicarbonate buffer (pH 8.1) for 1 h at RT on orbital shaker (190 rpm). The biotinylated phages were ultrafiltered on Amicon (30 kDa) by centrifugation (30 min, 15 000 g, 4°C), washed twice with carbonate-bicarbonate buffer, eluted and adjusted to initial concentration of ~10^8^ pfu/ml. Phages were serially diluted (two-fold from 1:2 to 1:128) in PBS supplemented with 0.05% Tween 20 (TPBS) and 0.1% of BSA (SERVA, Heidelberg, Germany) and added (100 μl) on LPS-coated microplate blocked previously for 1 h with 300 μl of 1% BSA in PBS (pH 7.4). After 1.5 h of incubation at 37°C, the plate was washed four times with TPBS and 100 μl of anti-biotin specific antibodies (conjugated with HRP) was added to each well. The plate was incubated for 1.5 h at 37°C, washed as described above and filled with 100 μl of TMB substrate (R&D, Minneapolis, USA). The plate was incubated 30 min at RT in the dark and finally the enzymatic reaction was stopped by addition of 50 μl of 1 M H_2_SO_4_. The absorbance was read at 450 nm (Asys UVM340, UK).

### Biofilm eradication by phages on peg-lid plates and characterization of phage-resistant biofilm isolates

Biofilms were formed on the polystyrene pegs surfaces protruding down from the lid (NUNC, Thermo Fisher Scientific Inc, Denmark) that fits into standard 96-well microtiter plates (NUNC, Thermo Fisher Scientific Inc, Denmark) according to the method described by Harrison *et al*. [25], with some modifications. Briefly, the pegs were placed in the wells of microplate containing 150 μl bacterial culture in tryptic soy broth (TSB; Becton Dickinson and Company, Cockeysville, MD) per well (~ 10^6^ cfu/peg). Biofilms were allowed to grow for different time periods (24 h up to 72 h) at 37°C without agitation and with a change of medium every 24 h during the whole duration of the experiment. Following the initial period of incubation, pegs were rinsed once with 0.9% NaCl to remove loosely adherent planktonic cells. Afterwards, the biofilm-containing pegs were transferred to 96-well plate in which 200 μl of the phages were prepared in TSB (10^8^ pfu/peg). Control experiments were performed under the same conditions. However the pegs (after immersion in NaCl) were inserted only in 200 μl TSB. The plates were incubated on a gyrorotary shaker at 125 rpm for 4 h at 37°C, after which the lids were removed and rinsed twice in 0.9% NaCl. To evaluate the biofilm-degradation properties of phages two assays were used: (i) cell viability assay and (ii) biomass in crystal violet (CV) assay [26]. All the assays were performed at least twice, with eight repeats for each. The results were presented as % of the untreated control samples. To determine the numbers of biofilm cfu/ml, the washed pegs were placed in a round-bottom microtiter “recovery” plate containing 200 μl per well of 0.9% NaCl. Biofilm cells were removed from pegs by sonication at 42 kHz in a water bath sonicator (POLSONIC model Sonic3, Poland) for 15 min. For each option tested, cells were collected from two parallel pegs, serially diluted in 0.9% NaCl and spot-plated onto TSA plates for colony counting.

In turn, to evaluate the biofilm biomass quantification, the rinsed pegs were fixed in 99% methanol for 15 min and air-dried. Next, pegs were placed into 96-well plate containing 250 μl per well of 0.01% CV (Aqua-Med, Poland) for 15 min at RT. After staining, pegs were washed seven-fold in tap water to remove all unbound CV and air-dried. The dye bound to the adherent biofilms was extracted with 200 μl 33% of glacial acetic acid, and the absorbance of the eluted dye in each well was measured by spectrophotometry at 595 nm (Asys UVM340, UK). To assess the emergence of phage-resistant variants of *P*. *aeruginosa* from the biofilms, several morphologically different colonies were recovered from 24 h, 48 h and 72 h biofilms treated with the phages. All the isolated colonies were cultured in TSB, and then the susceptibility of each isolates to the ancestral phages used in this study was determined using the spot assay. Furthermore, the ability to form plaques of cell-free supernatant recovered from wells was also examined. The experiments were carried out both after immediately isolation of persistent cells from the biofilm and their three successive passages on TSA to determine the stability of phenotypes.

### Phage influence on biofilm characteristics covering PET membrane

For these experiments three *P*. *aeruginosa* strains were used: PAO1 as reference strain, 0038 clinical strain (isolated from wound infection) from the collection of the Institute of Genetics and Microbiology, University of Wroclaw, Poland, and 708 clinical isolate (from cystic fibrosis patient) from the collection of the Prague CF Centre, Czech Republic. The analysis of biofilm degradation by live and UV inactivated phages was performed by microbiological methods as well as biophysical techniques. The biofilm was formed for 24, 48 and 72 h at 37°C in TSB medium on PET membrane with pore diameter 1 μm, as an element of BD Falcon Cell Culture Inserts. Biofilm was washed three times by fresh TSB medium and incubated for 4 h with phages (5×10^8^ pfu/ml) at 37°C. After incubation, the efficacy of biofilm degradation was tested using CV staining [26], spectrophotometry and fluorometry analysis of pyocyanin [27] and pyoverdin secretion [28], respectively. The laser interferometry [29–33] and goniometry analyses of growth medium diffusion through biofilm matrix was performed only for PAO1 strain. The above methods are described in S1 Text file.

### Phage DNA isolation

Phage DNA isolation was performed with the modified protocol for λ DNA isolation as previously described [13]. Purified phage particles (10^10^ pfu/ml) were disrupted by 1 h incubation at 56°C in 0.5% (w/v) SDS (Janssen Chimica, Geel, Belgium), 20 mM EDTA (Acros Organics, Geel, Belgium) and 5 μg/ml proteinase K (Thermo Fisher Scientific Inc, MA, USA). After a phenol/chloroform DNA extraction and ethanol precipitation, an additional RNase A treatment (100 μg/ml; Roche Applied science) was required to remove residual RNA. The genome of bacteriophage KT28 and KTN6 were deposited at GenBank under accession number KP340287 and KP340288 respectively.

### Phage genome sequencing

High throughput sequencing was performed on isolated phage DNA using the Illumina MiSeq platform available at the Nucleomics Core (VIB, Belgium). A 2*150 bp paired-end library (Nextera XT sample prep) was prepared and sequenced. The reads were assembled in a single contig with a 100–6000 fold coverage using CLC genomics Workbench *de novo* assembly algorithm (CLC bio, Cambridge, MA).

### *In silico* analysis

Potential ORFs were identified using the GeneMark S [34] and manually analyzed. Translated ORFs were compared to known proteins using BLASTP [35], the HHpred server [36,37] and HMMER [38], providing further insight into the predicted function of proteins. Conserved protein domains were identified using the Pfam [39]. Putative tRNA genes were searched for using the tRNAscan-SE program [40]. The intergenic regions were screened for regulatory elements using MEME [41], PHIRE [42] and manually curation. Putative factor-independent terminators were identified with ARNOLD software [43].

### Network construction, analysis, and assignment of phages into the clusters

To construct a protein-sharing network of KTN6 and KT28, each predicted protein was clustered into protein family using the ACLAME database (version 0.4) [44] with the database of “viruses” (as of November 2014) and an E-value <0.001 [45]. In addition, for the phages that share high gene contents with KTN6 and KT28, but absent in the ACLAME database, 918 protein sequences were retrieved from JG024 (NC_017674), PB1 (NC_011810), 14–1 (NC_011703), NH-4 (NC_019451), SPM-1 (NC_023596), LMA2 (NC_011166), LBL3 (NC_011165), KPP12 (NC_019935), SN (NC_011756), and ECML-117 (JX128258) and analyzed in the same manner as previously described [46]. Further, the phage-phage similarity for each of 12 phages was determined, based on the number of shared protein families with other phages using the hypergeometric formula. With a stringency threshold of similarity score >1 [45] the network was generated, that consists of 433 nodes representing phages and 6,366 edges representing the relationships between nodes. The network was visualized with an edge-weighted spring embedded layout using Cytoscape 3.1.1 (http://cytoscape.org/). Network properties were calculated with the Network Analyzer plugin [47]. In addition, nodes were assigned into the clusters using an algorithm determined by Lima-Mendez *et al*. [45]. Finally, phages that did not show any connections to such phage candidates were excluded for clarity.

## Results

### Isolation and morphology

**Fig 1:**
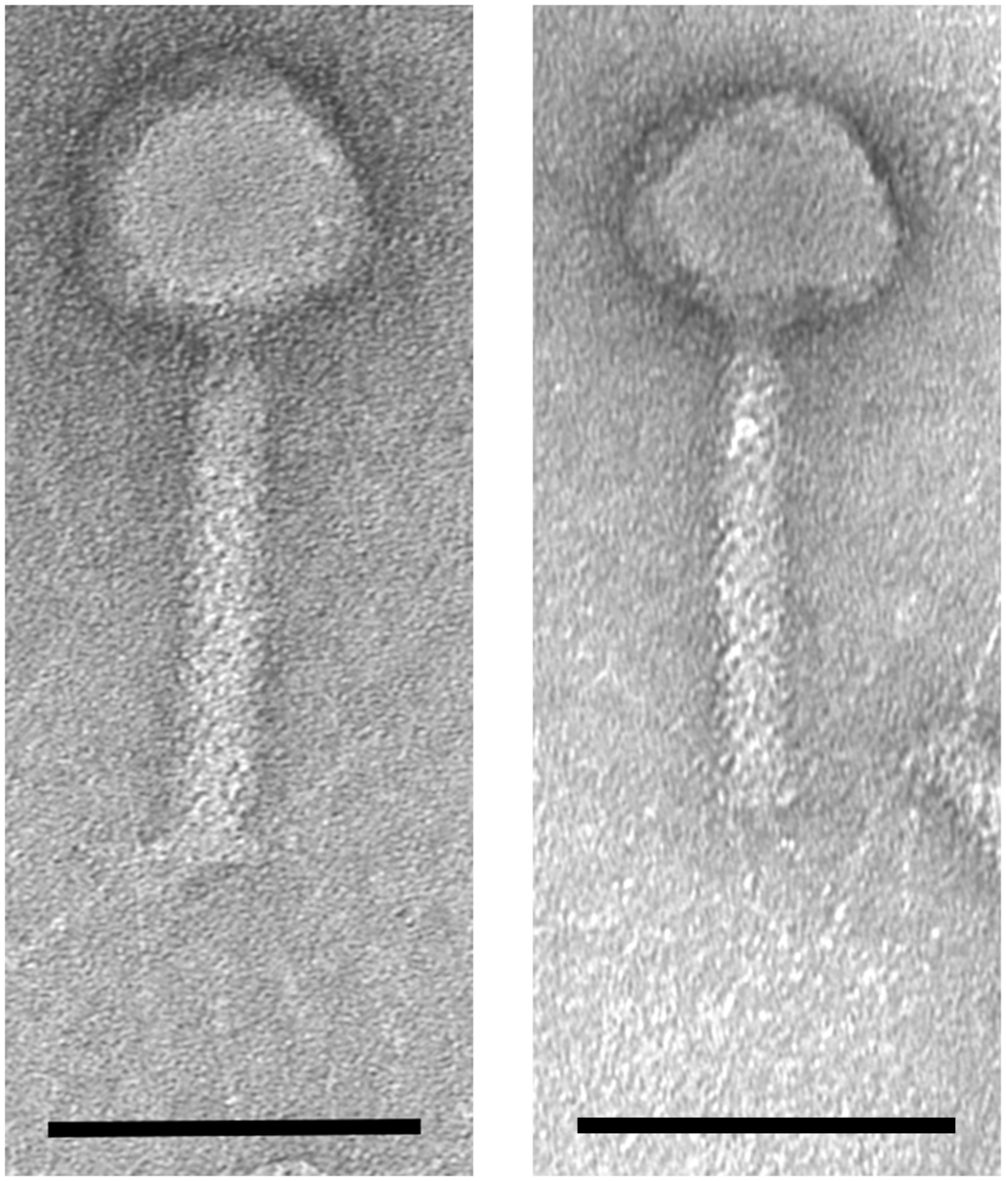
Transmission electron microscopic images of phage KT28 and KTN6. (A) KT28 has a isometric capsid with a diameter of 74 nm between opposite apices, a contractile tail 136 nm with a base plate. (B) KTN6 has a isometric capsid with diameter of 72 nm between opposite apices and a contractile tail 123 nm with a base plate. The scale bar represents 100nm.

Two *Pseudomonas* lytic phages were isolated from sewage samples collected in natural waste-water treatment plant (irrigated fields) located in Wrocław, Poland. After purification phage titres were 10^9^–10^10^ pfu/ml and caused 1.2–1.5 mm wide clear plaques with halo zone on 0.6% soft agar. The bacteriophages were examined by transmission electron microscopy (TEM) and classified on the basis of their morphological features to *Pbunalikevirus* (order *Caudovirales*, family *Myoviridae*) (Fig 1). The isolates were named as vB_PaeM_KT28 (KT28) and vB_PaeM_KTN6 (KTN6). The isometric head size was estimated to be 74 nm and 72 nm between opposite apices, and contractile tails 136 nm and 123 nm in the extended state, for KT28 and KTN6, respectively (S2 Table).

### One-step growth and stability tests

One-step growth experiments indicated a latent period of 35 min and a burst size of about 64 ± 0.98 and 96 ± 0.2 phage particles per infected bacterial cell, for KT28 and KTN6, respectively (S2 Table). The stability to physical and chemical agents of both phages was very similar (S2 Table). No reduction of pfu/ml was noticed over a period of 60 min at a temperature of 40–70°C, while more than 90% phages lost their activity after 15 min incubation at 80°C. Infectivity was not affected by chloroform at a concentration of 50% after 1h incubation at RT and at 4°C. Phage particles were relatively stable within a broad pH range. After 1 hour incubation at RT at pH between 3 and 12, over 99% phages were alive.

### Determination of phage receptor and host range

Phage typing showed, that phage KTN6 exhibited a broader spectrum of activity against tested *P*. *aeruginosa* strains, lysing 67%, compared to 59% for phage KT28 (S1 Table). This finding was also confirmed during phage typing of an international *P*. *aeruginosa* panel collection described by De Soyza *et al*. (2013) [48]. The KTN6 and KT28 propagated on 42% and 28% of the panel strains, respectively [in submision]. Moreover, both phages were more active compared to two Pb1-like phages: LMA2 24.14% and LBL3 45.55% (S1 Table). Jarrell and Kropinski (1977) [15] showed that PB1 and its relatives use the bacterial LPS layer as a receptor. In our study with PAO1 mutants, we also confirmed that phage KT28 and KTN6 recognize the LPS elements as its specific receptor, and only absence of A- and B-band O-antigen inhibits phage propagation. These results were confirmed by the experiments performed with pure LPS isolated from strain PAO1. The phage inactivation assay revealed direct correlation between LPS concentration and viral particles infectivity inhibition (S1 Fig), and that 36.0 μg/ml and 43.3 μg/ml of LPS was needed to inhibit the activity of 50% of 3 × 10^3^ pfu, for KT28 and KTN6, respectively (S2 Table). The LPS-binding experiments based on biotinylated phages showed similar OD values for both phages (0.74 and 0.75) (S2 Table).

### Biofilm eradication measured on peg-lid plates and characterization of phage-resistant biofilm isolates

Biofilm degrading activity of the tested phages was evaluated on peg-lid plates by measurement of colony forming units surviving phage treatment and biofilm mass value detection by crystal violet staining. It was shown that neither active nor inactivated phages had effect on biofilm mass eradication in 24, 48, and 72h culture, although significant reduction in living cells was noticed when active phages were applicated (S2 Fig). The KT28 and KTN6 infective particles were able to reduce 70–90% of the bacterial cells hidden inside the biofilm structure, regardless of culture age. Surprisingly KT28 inactivated particles appear equally efficient in cfu reduction as active viruses on 48h biofilm culture. The experiments regarding the emergence of phage-resistant variants of *P*. *aeruginosa* from the biofilms revealed that after just one day of active phages KT28 and KTN6 treatment, above 90% of persistent *Pseudomonas* cells were unsensitive to both phages and it was cross resistance. This feature was relatively stable because even three consecutive passages on TSA plates did not restore the susceptibility to tested phages. The cell-free supernatant recovered from wells exhibited the same activity as primary phage preparations suggesting no phage mutants generation during co-existence with resistant host variants. The persistent bacteria differed in morphology (small colony variants) comparing to parent phage-sensitive clones.

### Effect of phages on biofilm analyzed by cultivation methods on PET membrane

**Fig 2:**
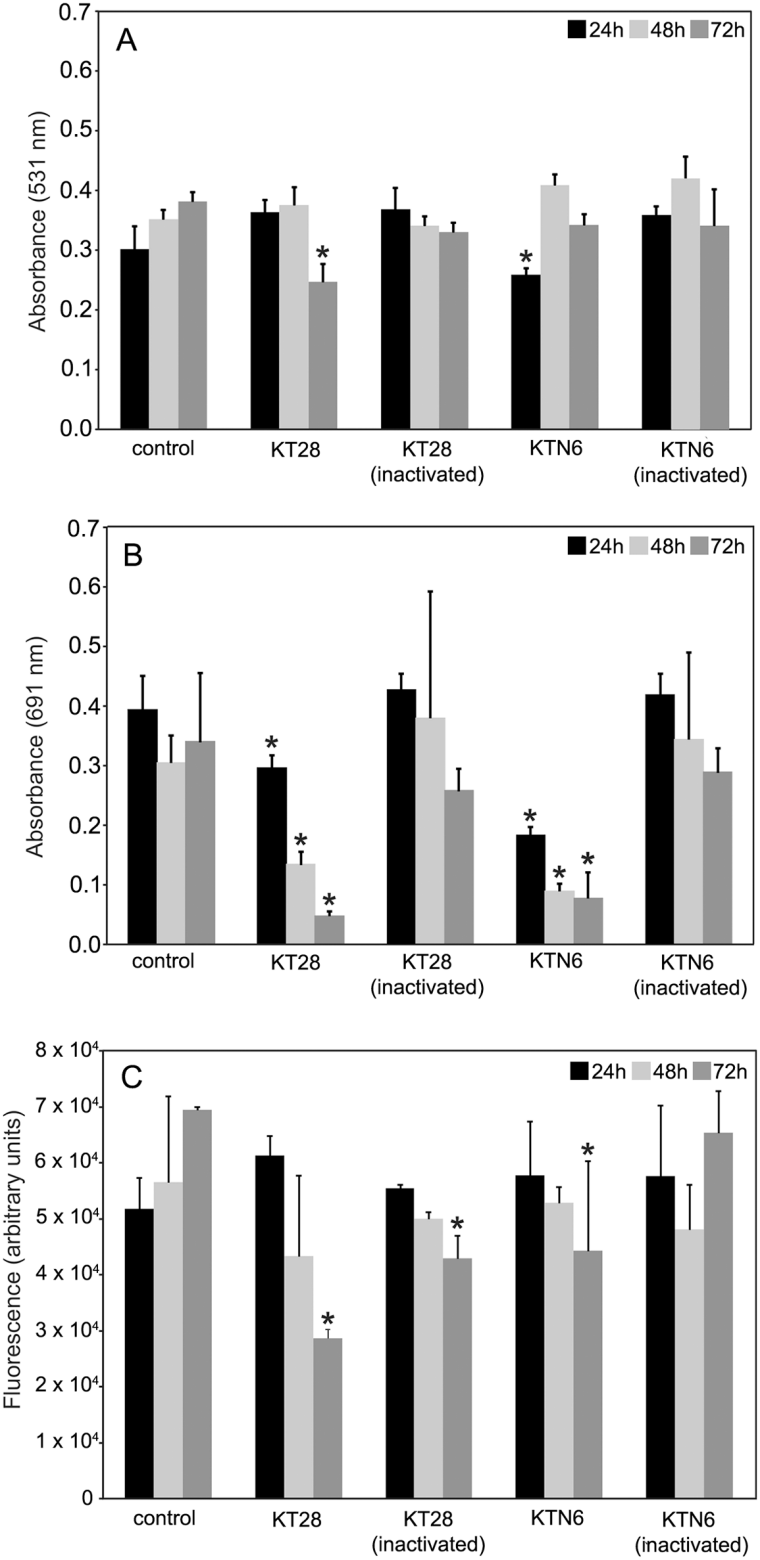
The effect of KT28 and KTN6 phage treatment on PAO1, 0038 and 708 strains biofilm formed on PET membrane. Biomass evaluation by CV staining (A,D,G); the level of pyocyanin in growth medium (B,E,H); the fluorescence of pyoverdin in growth medium (C,F,I). Untreated biofilm was used as control. The results are presented as the means ± SD. Statistical analysis was made by the ANOVA test (denoted p-values).

The effect of KT28 and KTN6 phages on biofilm formed on PET was determined by CV staining as well as pyocyanin and pyoverdin production (Fig 2). The analysis of biofilm mass eradication showed significant effect on 72 h biofilm of all three strains for active KT28, and 24 h (PAO1) and 72 h (clinical 708) biofilms for active KTN6 (Fig 2A, 2D and 2G). No biofilm mass eradication was noticed for inactivated phages. There, the observed differences between two techniques of biofilm mass detection (peg lid plates and on PET membrane) suggest a lower sensitivity for the peg technique. Analysis of pyocyanin concentration in growth medium showed that both active phages significantly reduce the level of this compound for all tested strains but on various biofilms (24, 48 or 72 h) in comparison to non-effective phages treated with UV radiation (Fig 2B, 2E and 2H). The second bacterial dye tested was pyoverdin serving as pathogenicity agent—a siderophore. The fluorescence of pyoverdin in the growth medium was significantly lower after incubation of PAO1 biofilm formed for 72 h with both active phages and inactivated KT28 (Fig 2C). Active KTN6 reduced the pyoverdin concentration for 72 h biofilm of both clinical strains, while KT28 only for 48–72 h biofilm of 0038 strain (Fig 2F and 2I).

### Effect of phages on biofilm disruption analyzed by biophysical techniques

**Fig 3:**
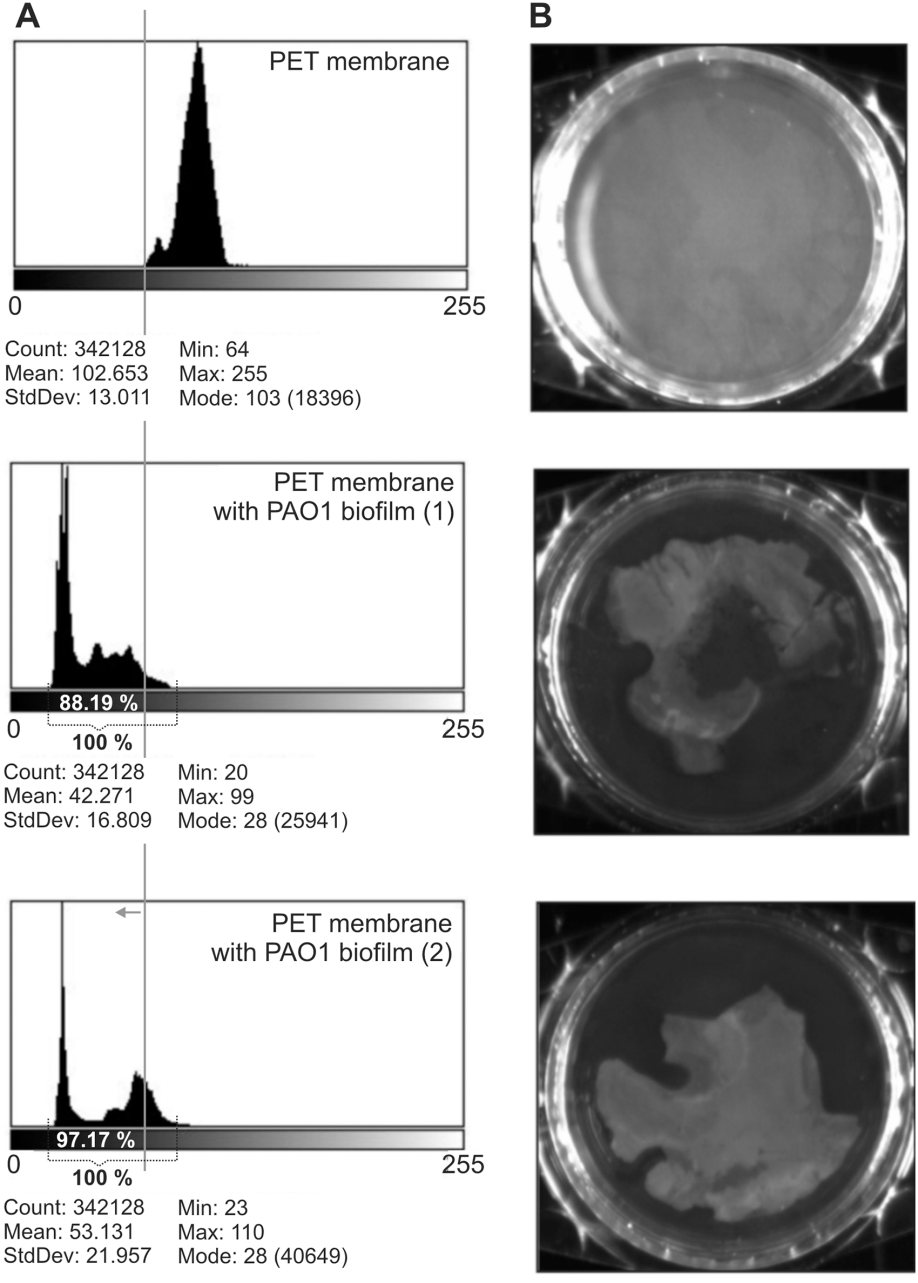
Histograms of imageJ software analysis of PAO1 biofilm (stained by CV) formed on PET membrane. (A) In left panel grey line presents cut-off point in which pixels from total analyzed (membrane with biofilm) have the grey level lower than control (native membrane). The % of membrane covering by biofilm formed for 72 h is presented. (B) Right panel presents images of analyzed membranes with/without biofilm. Analysis were done in two independent experiments.

**Fig 4:**
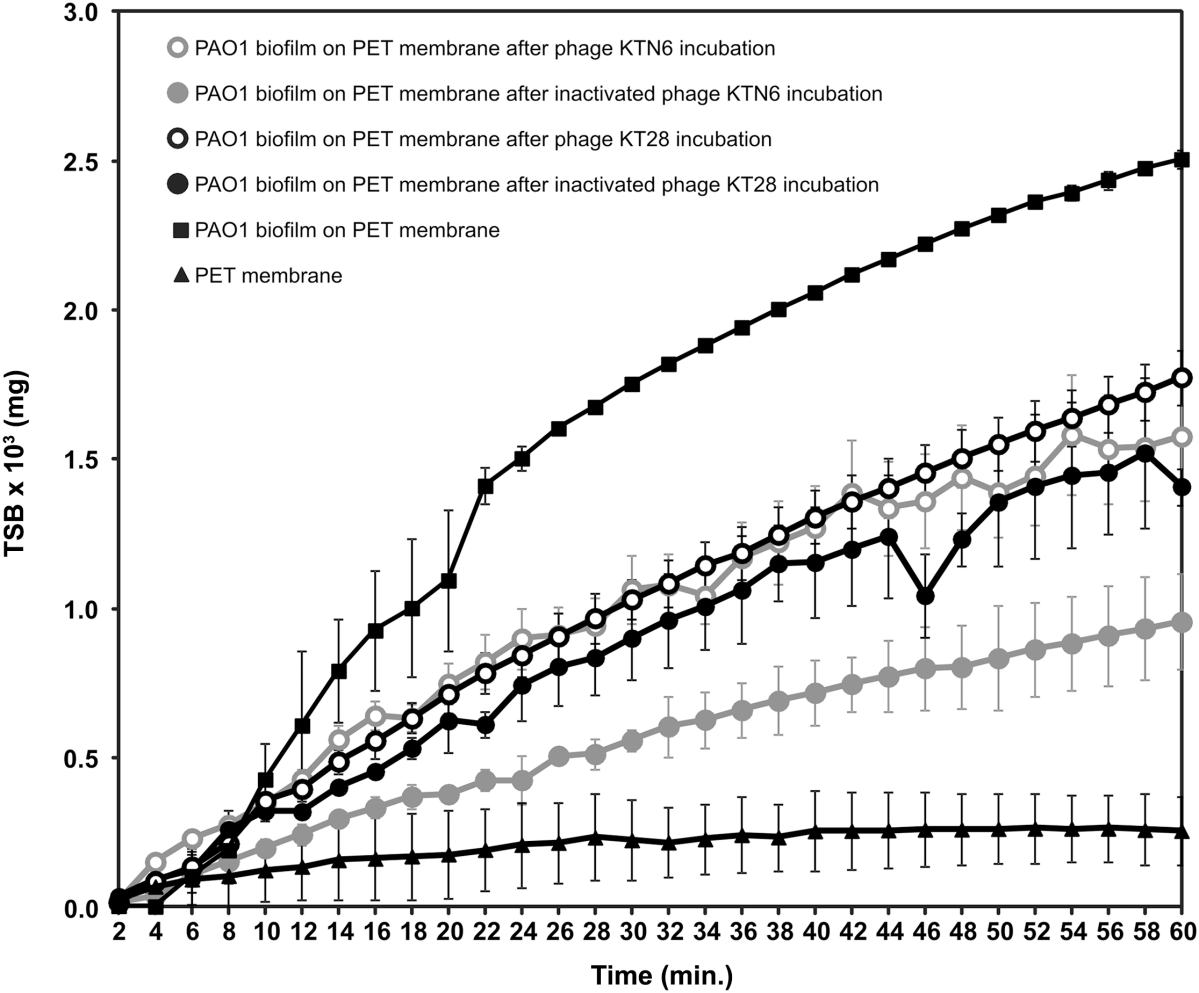
Laser interferometry analysis of TSB medium diffusion through PAO1 biofilm treated with phages. Untreated biofilm was used as control. The results are presented as the means ± SD from three independent experiments.

**Fig 5:**
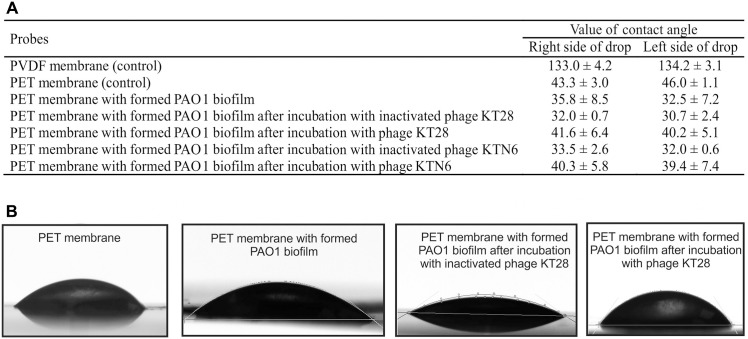
The goniometry analysis for TSB medium drop on PAO1 biofilm surface on PET membrane. (A) The value of contact angle. (B) The images of TSB drops on membrane surface. Native membranes were used as control. The results are presented as the means ± SD from three independent experiments.

The increase of TSB diffusion through the biofilm might indicate its structure disruption. ImageJ software analysis of membrane images with biofilm (degree of membrane covering by biofilm; %) showed that the highest degree of PET membrane covering by PAO1 biofilm was obtained after 72 h (92.58% ± 6.35) (Fig 3). We decided to use PET membrane with a biofilm which was formed for 72 h for laser interferometry analysis. Fig 4 shows the laser interferometry analysis of growth medium diffusion through PAO1 biofilm treated with KT28 or KTN6 phage (active and inactivated) for 4 h. Native PET has hydrophobic properties and diffusion of water-soluble compounds (as TSB medium) through this membrane was limited. The amount of TSB medium transported through PET (2.56×10^–4^ mg/h) was statistically lower than through membrane with hydrophilic biofilm (2.51×10^–3^ mg/h) (p<0.001). After the biofilm incubation with four types of tested phages, and the amount of transported medium was lower compared to untreated PAO1 biofilm (p<0.001). This correlates to biofilm degradation. This indicates that degradation of biofilm structure by phages might be associated with: (i) the loss of biofilm matrix elements on membrane surface and the reversion to hydrophobic properties of membrane, or (ii) partial degradation of matrix/biofilm structure resulting on higher diffusion rate. This observation was partially confirmed by goniometry analysis (Fig 5). The value of contact angle for uncovered PET membrane (43.3) was similar to the angle value of membrane covered by biofilm and treated with both active phages (41.6 for KT28 and 40.3 for KTN6). The inactive form of phages gave more hydrophilic feature of membrane with the angle value equal to non-treated biofilm suggesting reduced eradication compared to active phages.

### Genome and comparative genome analysis

**Fig 6:**
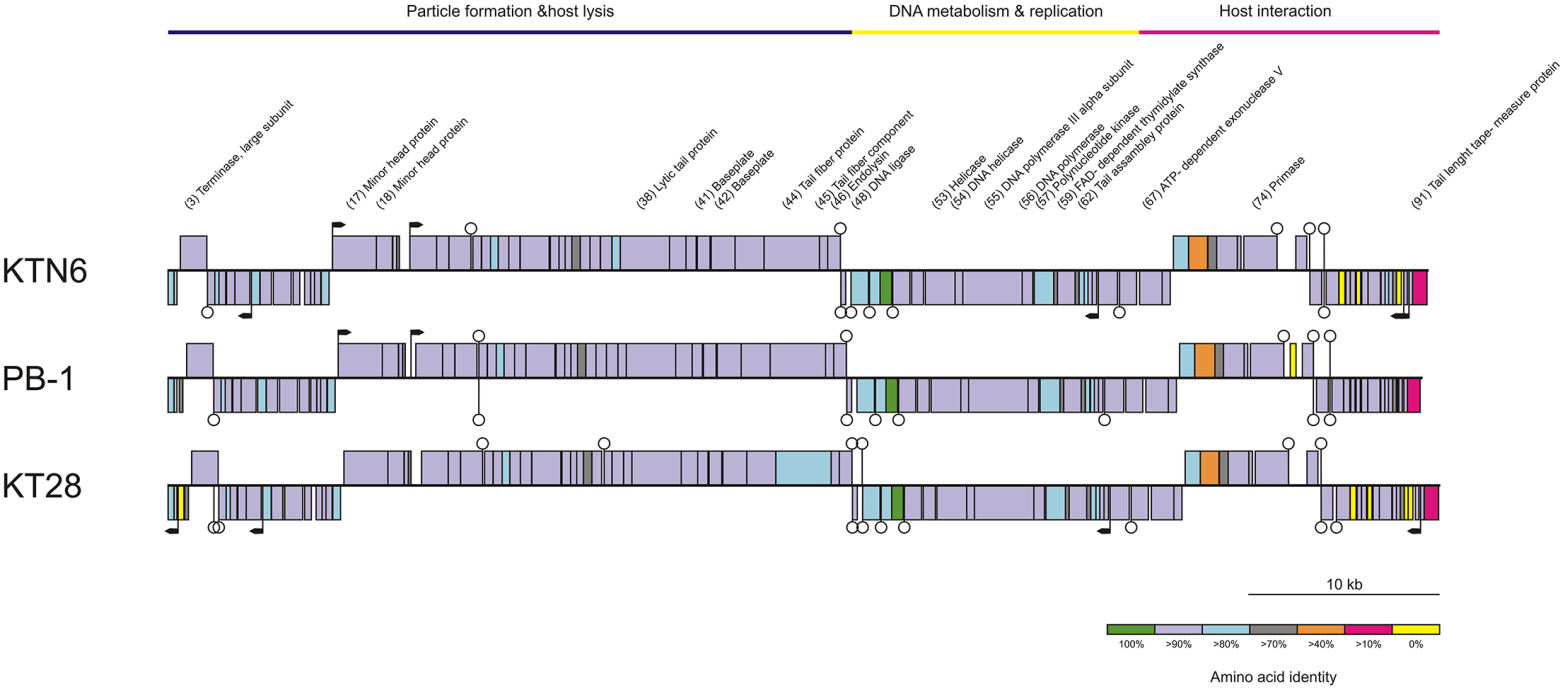
*In silico* analysis of KTN6 and KT28 phages and their comparison to PB1 phage. The predicted open reading frames of presented phages (rectangles) and their amino acid identity to the corresponding ORF in PB1 phage, indicated with different colors in the legend. Predicted terminators and promoters are shown as stem-loop structures and black arrows, respectively.

Using high throughput sequencing by the Illumina MiSeq platform, the complete genome sequences were determined (Fig 6, S2 Table). Phages show 96.43% nucleotide similarity to each other, but when compared to other Pb1-like phages (14–1, BcepF1, F8, LBL3, LMA2, PB1, SN) they display nucleotide similarity between 47.74–96.19% for KT28 and 46.94–96.55% for KTN6 (S3 Table), with most similarity to LMA2, 96.19% and 96.55%, respectively. BcepF1 phage showed a more distant relationship to Pb1-like phages, showing only 47.74% and 46.94% similarity to KT28 and KTN6, respectively and also below 48% to the other tested phages. When comparing *in silico* the protein identity of these new phages to PB1, 71% of the proteins showed more than 90% similarity, especially in the regions responsible for particle formation, host lysis, and DNA metabolism and replication (Fig 6). It confirms that the main core of Pb1-like phage genomes is very conserved. The largest variation of proteins was observed at the beginning and the end of the genomes. The tRNAscan-SE program did not reveal tRNA in genomes. We were also able to identify 15 putative factor-independent terminators in KT28 and 12 in KTN6 as well as 4 and 5 promoters, respectively (Fig 6). GC (%) was 55.6 for KT28 and 55.51 for KTN6.

**Fig 7:**
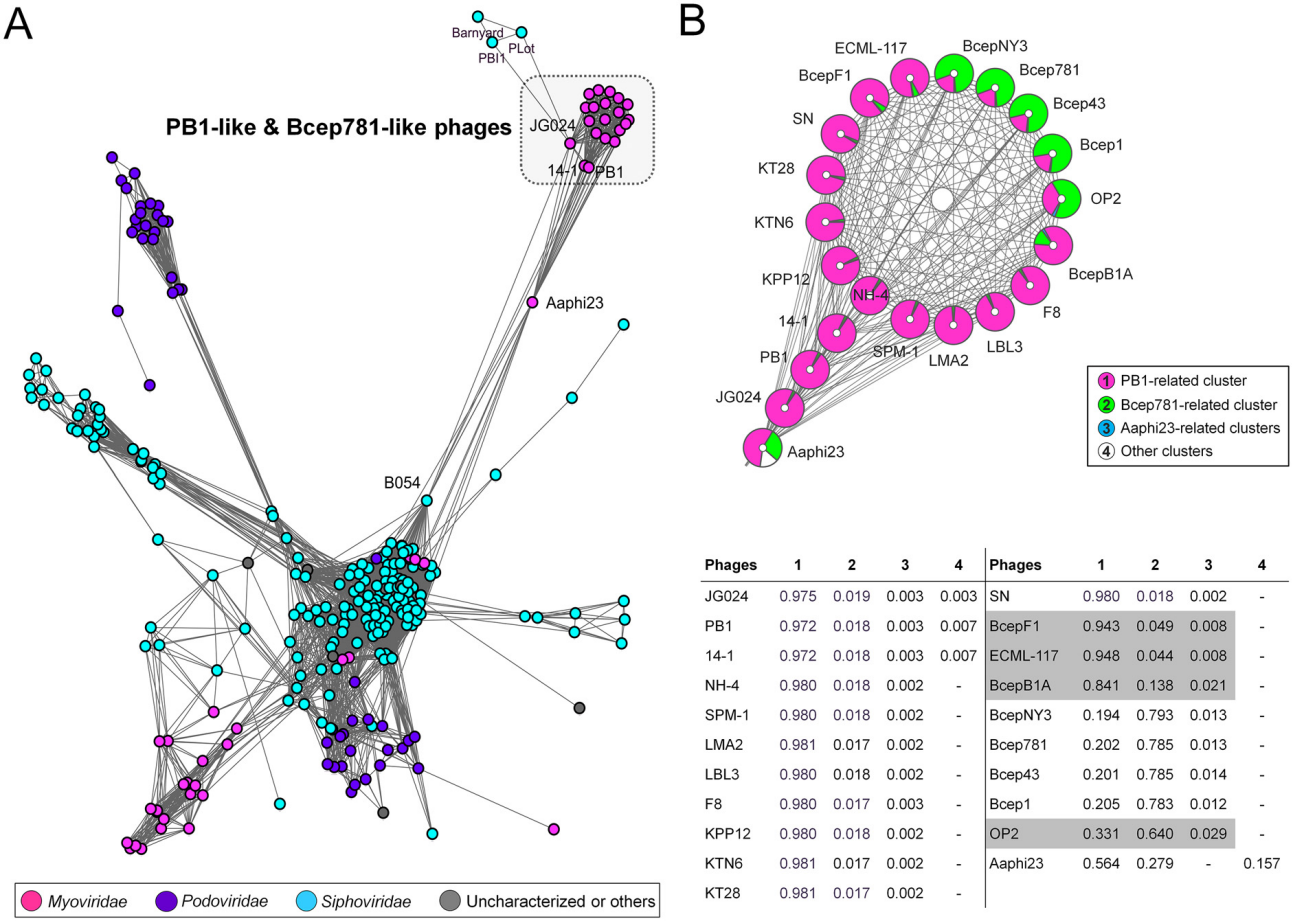
Protein-sharing network and reticulate relationship of PB1-related and Bcep781-related phage populations. (A) A network representation was produced using the edge-weighted spring embedded layout of Cytoscape version 3.1.1. Nodes indicate phages and edges between two nodes indicate their statistically weighted pairwise similarities with significance (Sig) scores of >1. In this way, 4,928 edges from 274 phages, including KTN6, KT28, and additional Pb1-like phage members (JG024, PB1, 14–1, NH-4, SPM-1, LMA2, LBL3, KPP12, and SN) are shown and 159 phages that did not show any connections to those candidate phages were excluded for clarity. Node color corresponds to a given ICTV taxonomic class of *Myoviridae*, *Siphoviridae*, and *Podoviridae*, and uncharacterized and other phages. (B) Assigning of the phage clusters onto population network composed of interactions among Pb1-like phages, Bcep781-like phages, Aaphi23, and others. Node pie charts show the proportion of homogeneity (membership) as estimated by estimating the proportion of the weight of connections to a given node to the nodes within a particular cluster. A color code for each cluster is represented in the legend box. Each membership value relevant to the represented phage is illustrated as the membership matrix in the lower panel.

Fig 7 shows a resulting network that consists of 274 phages belonging to *Myoviridae*, *Siphoviridae*, *Podoviridae*, or uncharacterized and another phages, and 4,928 relationships between them. In the network, KTN6, KT28, and other 13 phage members belonging to Pb1-like group, together with Bcep781-like phages, were mostly placed into a small component at the periphery (Fig 7). Aaphi23 bridged such component comprising two phage groups with the largest component, whereas B054 linked to JG024, PB1, and 14–1; both of which are temperate phages [49,50]. In addition, JG024 showed connections with the two mycobacteriophages PBI1 and PLot.

To validate their positioning in the network, we used two classical population topologies such as the clustering coefficient (the extent to which the neighbors of a given node are interlinked) and the betweenness (a centrality estimating the proportion of shortest paths that pass through a node) [47]. We found that with the highest clustering coefficient of 1 (1 means absolute cohesiveness) (S4 Table), Pb1-like phages including KTN6, KT28, F8, NH-4, SPM-1, LMA2, LBL3, KPP12, SN, ECML-117, BcepF1, and BcepB1A were tightly interconnected with Bcep781-like phages, implying their closest evolutionary relationship. However, compared to Pb1-like members of a given component, JG024, PB1, and 14–1 showed lower clustering coefficients and higher betweenness distributions due to their more connected parts. Aaphi23 can be characterized by one of the highest betweenness node in the network.

Furthermore, we retrieved the reticulate clusters of Pb1-like and Bcep781-like phages with the methodology by Lima-Mendez *et al*. (2008) [45], in which the weight of intracluster connection of a phage, called the membership, is used to differentiate cluster and membership >0.79 can be a rough estimate of vertical evolution. In this reticulate classification, most phage members were highly assigned to their respective ICTV genera with membership >0.97 **(** Fig 7). Notably, BcepF1, ECML-117, and BcepB1A showed lower memberships to PB1 cluster ranging from 0.841 to 0.948, which is consistent with their marginal relationships to Pb1-like phages [14,51]. Also, OP2 displayed a lower membership to Bcep781 cluster = 0.640 and thus more divergent among Bcep781-like members.

## Discussion

The virion of newly isolated KT28 and KTN6 show characteristic features of *Pbunalikevirus* with respect to head/tail size and LPS host receptor recognition (S2 Table). Comparing both phages, the activity of KT28 virions was inhibited by a smaller amount of pure LPS with similar value of LPS binding. The KT28, with a larger genome encapsulated in a bigger virion, exhibits the same latent period, but much lower burst size in comparison to KTN6 phage (64 ± 0.98 pfu/cell versus 96 ± 0.2 pfu/cell). Both phages possess similar susceptibility to chemical and physical agents such as high temperature, chloroform and pH. Phage infectivity tested on clinical strains revealed that KTN6 was more potent than KT28, propagating on 67% versus 59% of the strains, respectively.

*P*. *aeruginosa*, an opportunistic pathogen has evolved a number of virulence features such as: (i) biofilm formation, (ii) secretion of factors that impair neutrophil phagocytosis and activation, (iii) type III secretion system-dependent cytotoxicity [52,53]. Antibacterial therapies using these newly isolated phages may target all the above features to reduce the severity of infection and increase the efficacy of immune response in bacteria eradication process. Biofilm eradication activity was tested on both peg-lid plate assay and PET membrane surface. It turned out that the first technique in biomass evaluation (CV staining) was not sensitive enough to detect any significant changes after phage treatment. In general, CV staining revealed unstable results. As a consequence, other methods were used to evaluate phage potency to affect biofilm forming bacterial cells. For this purpose, a colony count technique was applied and the analysis of pyocyanin and pyoverdin secretion by spectrophotometry and fluorometry was used. Furthermore, laser interferometry and goniometry were applied for the growth medium diffusion through biofilm matrix. The standard cfu evaluation revealed significant reduction (70%-90%) in 24–72 h old biofilm cultures for both Pb1-like phages, suggesting that phage particles were able to penetrate the biofilm matrix. However, the treatment of these phages caused the emergence of phage-resistant variants of *P*. *aeruginosa* from the biofilms. The persistent cells turned out to be cross resistant to both phages, probably by the loss or changes in O-antigen structure. This feature was relatively stable and persistent bacteria had small colony variant morphology. It may put the applicability of Pb1-like phages into question, although such enveloped modification may also decreases the fitness of the microbe and made them more susceptible to immune system defense.

*P*. *aeruginosa* produces highly diffusible pigmented toxic secondary metabolites, including a blue pigment, pyocyanin, that kills mammalian and bacterial cells through the generation of reactive oxygen intermediates and suppression of the acute inflammatory response by pathogen-driven acceleration of neutrophil apoptosis and by reducing local inflammation. This compound also functions as signaling molecules in the up-regulation of quorum sensing-controlled genes during stationary phase [54–56]. The ability of the phages to reduce the production of pyocyanin, supports the idea of bacteriophage application in severe infections caused by opportunistic pathogens. In our experiments, both active phages showed strong inhibitory activity on *P*. *aeruginosa* biofilm, regardless of the bacteria origin (laboratory, clinical isolate from wound infection and clinical isolate form CF patient).

Another compound secreted by *P*. *aeruginosa* is pyoverdin, serving as a siderophore competing directly with transferrin for iron significantly reducing the amount of bioavailable iron in infected individuals [57]. The tested phage KT28 was able to reduce the amount of pyoverdin secreted by PAO1 strain and clinical isolate 0038. The KTN6 phage affecting only 72 h old culture in active form but of all three tested strains. Our current investigation (data not published yet) are focused on correlation between bacterial cells growth and biofilm formation of *P*. *aeruginosa* strains, where the kinetics of bacterial growth (CFU and A_600 nm_) is positively correlated with pyocyanin level in supernatants, but not with biofilm mass stained by CV. It indicates that reduction of pyocyanin might be associated with dying of bacterial cells in the presence of tested phages. Moreover, pyocyanin and pyoverdin are accumulating in matrix during its formation and the final level in the mature biofilm might differ even for the same strain (the value of error bars). The reduction of released molecule of *Pseudomonas*-specific dyes could be correlated with phages activity. Statistically significant (p<0.005) inhibition observed for pyocyanin and pyoverdin production was evoked by phages (lysis of cells in biofilm). Summarizing both newly isolated Pb1-like phages were able to reduce the number of pseudomonads cells living in a biofilm structure. Moreover, the amounts of the most important dyes secreted by the pathogen (pyocyanin and pyoverdin) decreased significantly, which may prove the potency of isolated phages to be applied as efficient antibacterial in *Pseudomonas* biofilm treatment. Further analysis, done in our study, including diffusion and goniometry experiments, support this statement. The increase of diffusion rate through the biofilm matrix after phage application, indicated the degradation of the biofilm structure associated with the loss of biofilm matrix elements on membrane surface and the reversion to hydrophobic properties of membrane, or partial degradation of matrix/biofilm structure. It should be emphasized that the increase of the diffusion was also obtain after the application of inactivated particles, suggesting that both tested phages KT28 and KTN6 are equipped with exopolysaccharide depolymerases responsible for phage particle spread within the biofilm matrix. The ability of phages to eradicate biofilm structure was also confirmed by goniometry analysis, where the value of the contact angle of a biofilm treated with both active phages was similar to the angle value for uncovered PET membrane.

Genome comparison showed that KT28 and KTN6 are highly related to the widespread and conserved Pb1-like viruses. This group of phages shows a high sequence similarity and limited horizontal gene transfer [18]. To investigate genome evolution of KTN6 and KT28, we used a mathematical model of protein-sharing network, extending to probable close relatives. A graphical representation of the network can reveal several evolutionary connections of Pb1-related genomes to other phage genomes. A major observation is that most of the *Pbunalikevirus* members were densely interconnected to those of the Bcep781-like group, as measured by their clustering coefficients. Such near-one clustering indicates the conservation of a core genome among those two groups [45]. However, more importantly, they did not form a single group due to multiple connections of JG024, PB1, and 14–1 of the Pb1-like group to the temperate phage B054 [49] or two mycobacteriophages PBI1 and PLot. Based on this observation, two homologous genes were observed which only exist in all six corresponding phages and their positions in the limited conservation regions of the genomes of JG024, PB1, and 14–1 [58]; ORFs 13 of JG024 and 14–1, and ORFs 94 of JG024 and PB1, and ORF90 of 14–1 (S4 Table). This may indicate that gene gain or loss in such variable regions could be important for their diversity, despite the limited lateral gene transfer in Pb1-like group [13]. Such interesting links are revealed through our evolutionary reconstruction of gene phylogenies [58]. In addition, another temperate phage Aaphi23, that shows some chimeric features [45,50], acts as a bridge for both Pb1-like and Bcep781-like groups. This reflects the tendency of temperate phage-mediated genetic exchange between strictly lytic phages, Pb1-like and/or Bcep781-like phages, and the rest of the phage population [45,59]. Collectively, together with a reticulate classification, our phage population network revealed that Pb1-like and Bcep781-like phages most likely have diverged through vertical evolution with some vertically inherited modules and their ancestral relationship might be beyond the genus level at the subfamily level.

## Supporting Information

S1 Fig The phage inactivation assay by pure LPS. (TIF)

S2 Fig Phage effect on cells viability (A) and biomass (B) in the biofilm of *P*. *aeruginosa* PAO1 formed on peg-lid plates. Data were expressed as the percentage of control in reference to untreated control samples (100%). All the assays were performed at least twice, with eight repeats for each.
(TIF)

S1 Table Phage activity comparison of four different Pb1-like phages: KTN6, KT28, LMA2, LBL3, on the basis of phage typing with *P*. *aeruginosa* strains from Military Hospital Nederoverheembeek, Brussels, Belgium [21]. (DOCX)

S2 Table Major features of characterized phages. (DOCX)

S3 Table The comparison of KT28 and KTN6 to 7 other Pb1-like phages (14–1, BcepF1, F8, LBL3, LMA2, PB1 and SN) based on nucleotide similarity. Percent Identity Matrix—created by Clustal2.1 (http://www.ebi.ac.uk/Tools/msa/clustalw2/).
(DOCX)

S4 Table Clustering coefficient and betweenness centrality in the protein-sharing network of phage Aaphi23, and Pb1-like/Bcep781-like phages. (DOCX)

S1 Text Materials and methods used for phage influence on biofilm characteristics covering PET membrane. (DOCX)
