# Correction: Characterization of the Newly Isolated Lytic Bacteriophages KTN6 and KT28 and Their Efficacy against *Pseudomonas aeruginosa* Biofilm

**DOI:** 10.1371/journal.pone.0137015

**Published:** 2015-08-25

**Authors:** Katarzyna Danis-Wlodarczyk, Tomasz Olszak, Michal Arabski, Slawomir Wasik, Grazyna Majkowska-Skrobek, Daria Augustyniak, Grzegorz Gula, Yves Briers, Ho Bin Jang, Dieter Vandenheuvel, Katarzyna Anna Duda, Rob Lavigne, Zuzanna Drulis-Kawa

The image for Fig 2 is incorrect. Please see the corrected Fig 2 here.

**Fig 2:**
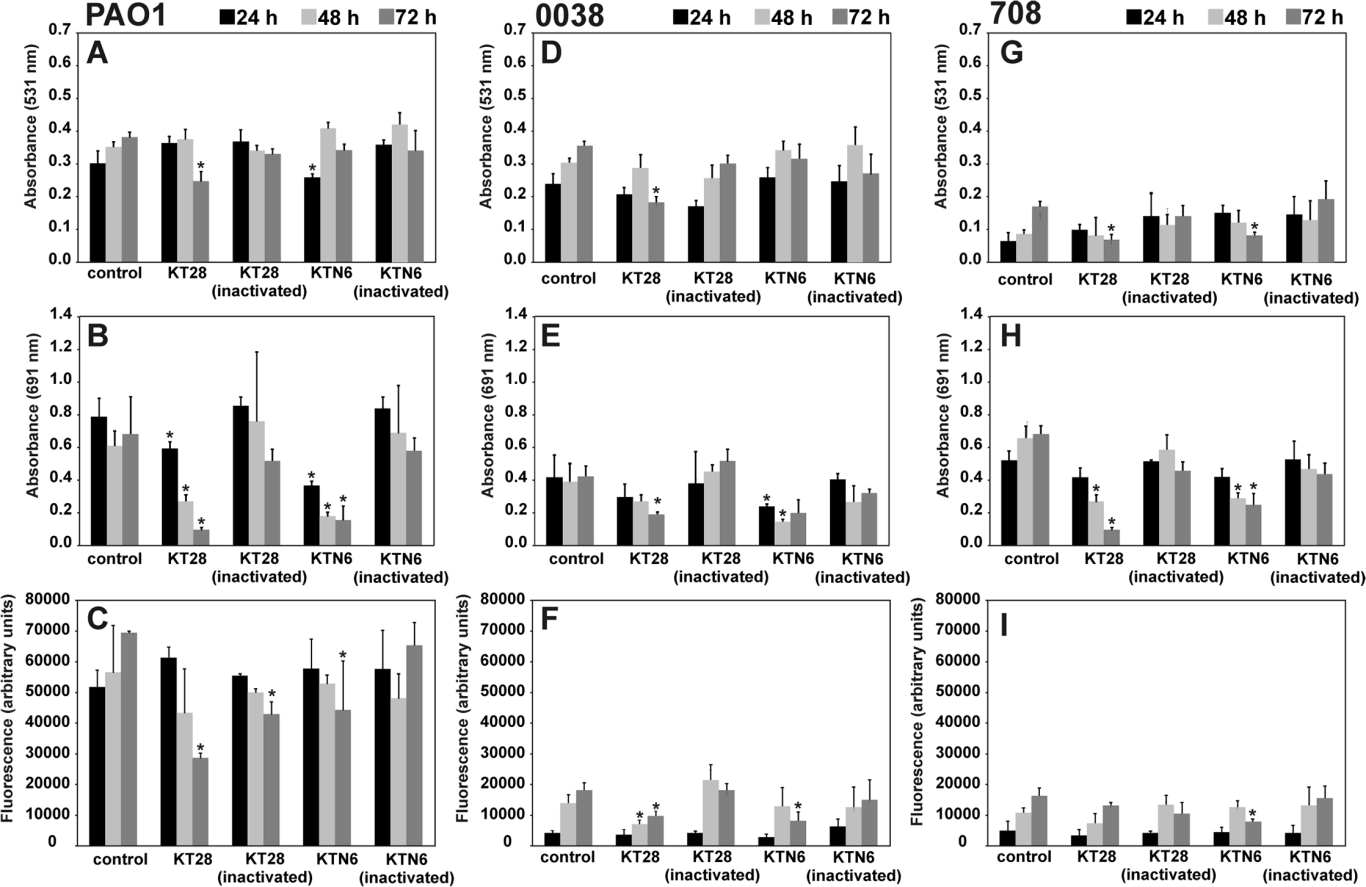
The effect of KT28 and KTN6 phage treatment on PAO1, 0038 and 708 strains biofilm formed on PET membrane. Biomass evaluation by CV staining (A,D,G); the level of pyocyanin in growth medium (B,E,H); the fluorescence of pyoverdin in growth medium (C,F,I). Untreated biofilm was used as control. The results are presented as the means ± SD. Statistical analysis was made by the ANOVA test (denoted p-values).

There are multiple errors in Fig 4. Please see the corrected Fig 4 here.

**Fig 4:**
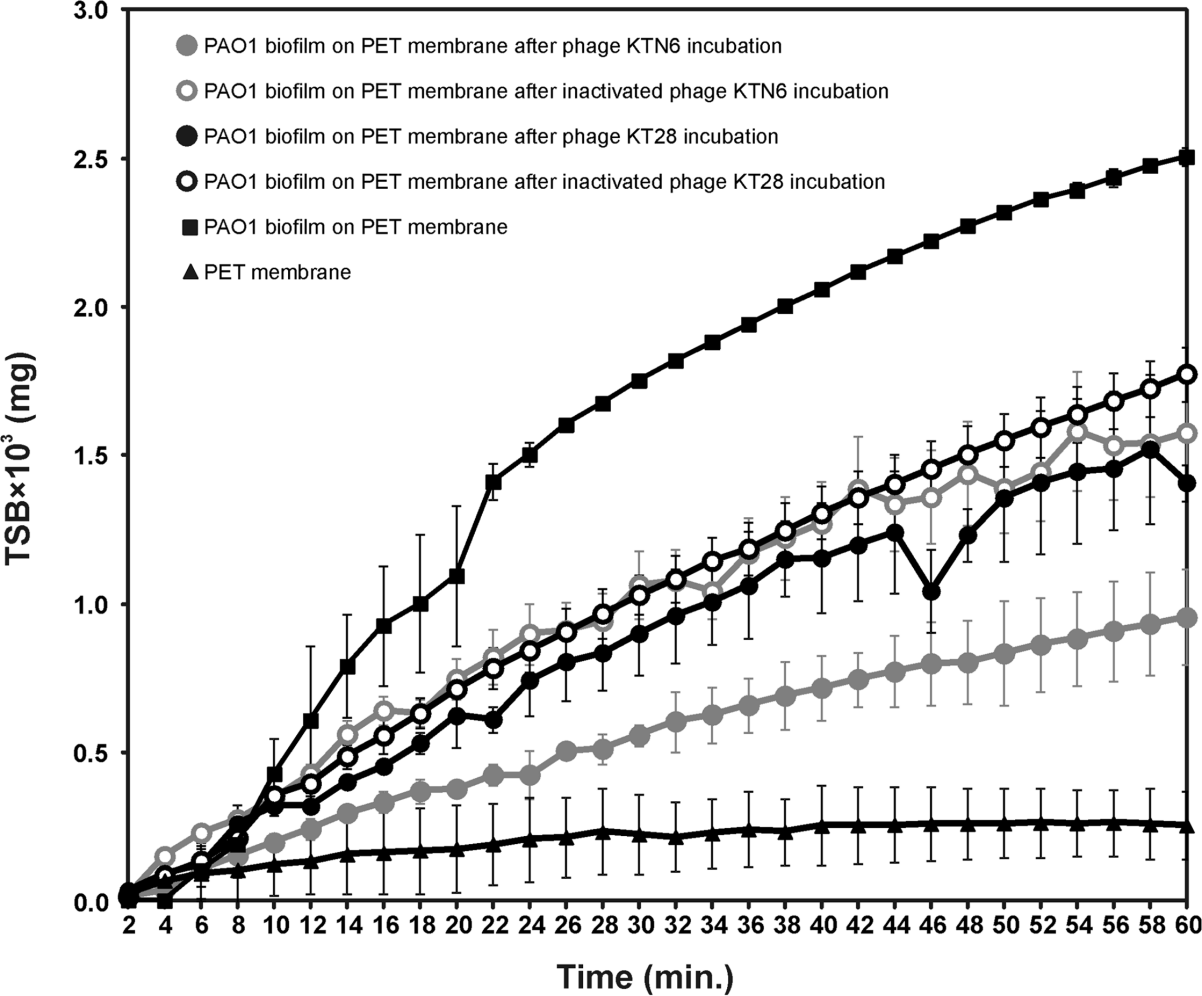
Laser interferometry analysis of TSB medium diffusion through PAO1 biofilm treated with phages. Untreated biofilm was used as control. The results are presented as the means ± SD from three independent experiments.
