# Supplementary figures and images for: Characterization of the Newly Isolated Lytic Bacteriophages KTN6 and KT28 and Their Efficacy against Pseudomonas aeruginosa Biofilm
Source: PLoS One. 2015 May 21;10(5):e0127603. doi: 10.1371/journal.pone.0127603 (PMC4440721; doi:10.1371/journal.pone.0127603)

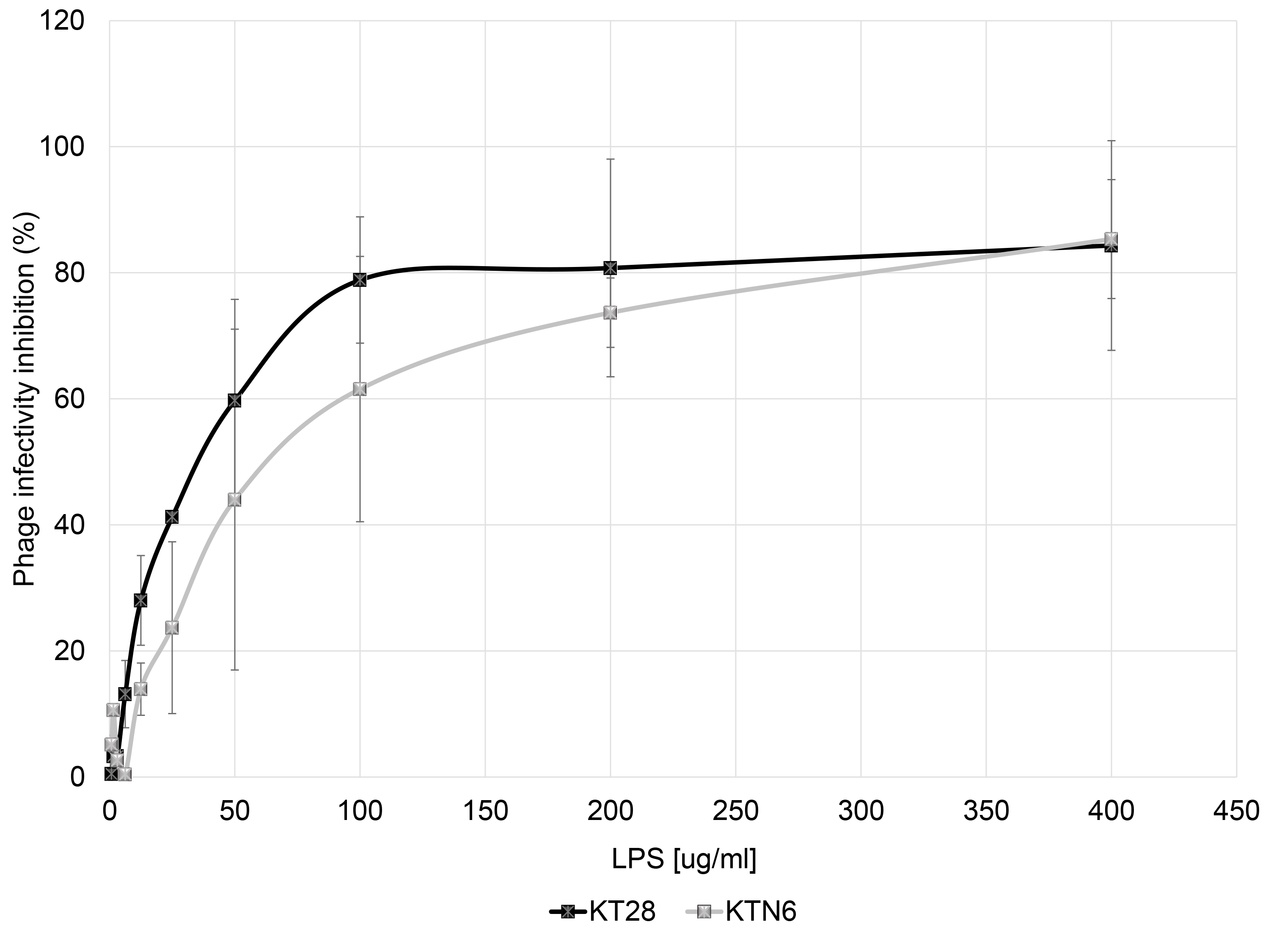

Supplement: S1 Fig — (TIF) [file pone.0127603.s001.tif]

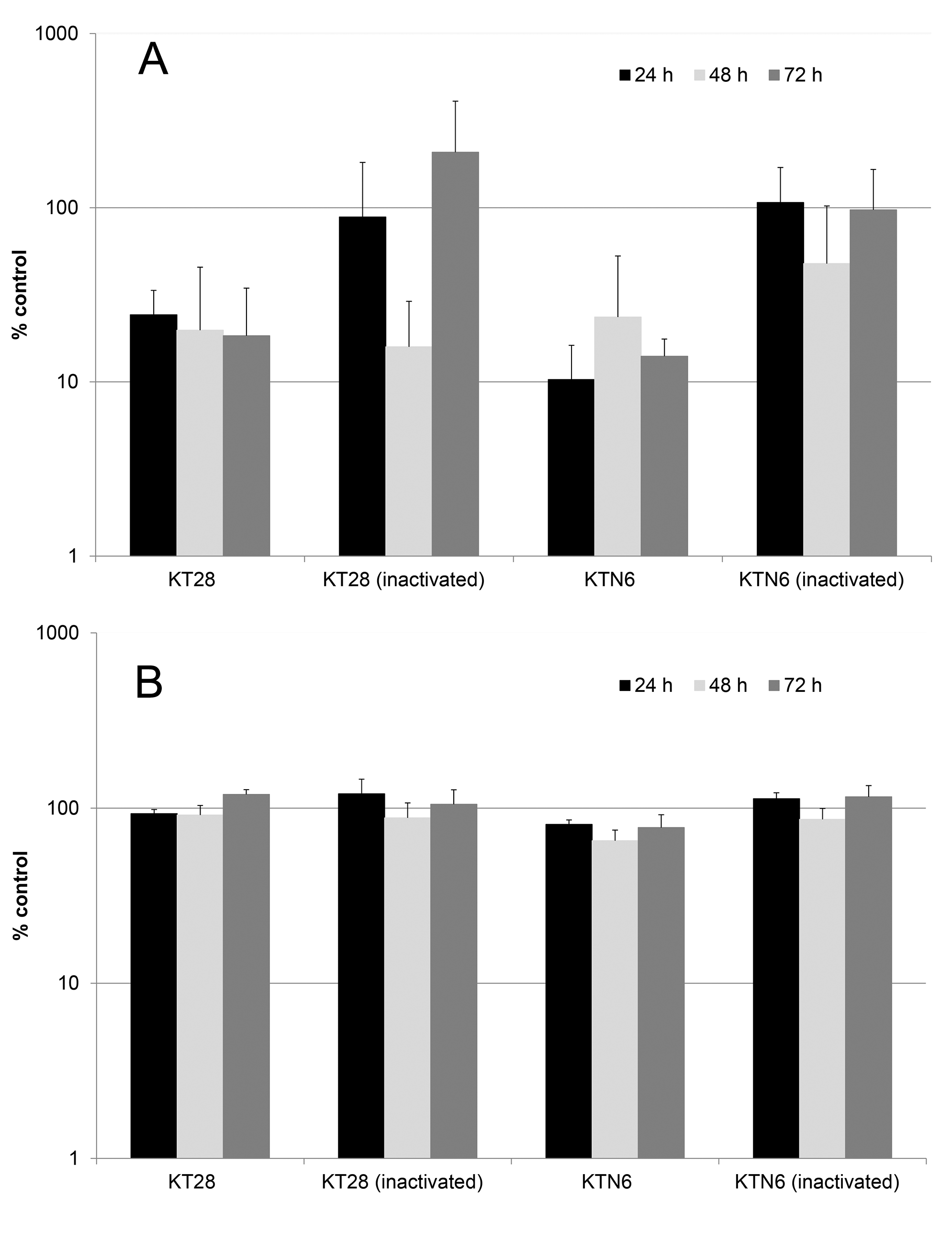

Supplement: S2 Fig — Data were expressed as the percentage of control in reference to untreated control samples (100%). All the assays were performed at least twice, with eight repeats for each. (TIF) [file pone.0127603.s002.tif]
