# Supplementary material for: Characterization of the Newly Isolated Lytic Bacteriophages KTN6 and KT28 and Their Efficacy against Pseudomonas aeruginosa Biofilm
Source: PLoS One. 2015 May 21;10(5):e0127603. doi: 10.1371/journal.pone.0127603 (PMC4440721; doi:10.1371/journal.pone.0127603)
Supplement: S1 Table — (DOCX) [file pone.0127603.s003.docx]

**S1 Table.** **Phage activity comparison of four different Pb1-like phages: KTN6, KT28, LMA2, LBL3, on the basis of phage typing with *P. aeruginosa* strains from Military Hospital Nederoverheembeek, Brussels, Belgium [21].**

|  | ***PA* strains** | **KTN6** | **KT28** | **LMA2** | **LBL3** |
| --- | --- | --- | --- | --- | --- |
| **1** | US449 |  |  |  |  |
| **2** | LMG14083 |  |  |  |  |
| **3** | Bu007 |  |  |  |  |
| **4** | PAO23 |  |  |  |  |
| **5** | Aa 249 |  |  |  |  |
| **6** | US448 |  |  |  |  |
| **7** | PAO1 Krylov |  |  |  |  |
| **8** | Lo050 |  |  |  |  |
| **9** | US450 |  |  |  |  |
| **10** | Li004 |  |  |  |  |
| **11** | Be128 |  |  |  |  |
| **12** | Lo053 |  |  |  |  |
| **13** | ATCC 27853 |  |  |  |  |
| **14** | Br906 |  |  |  |  |
| **15** | PhDW6 |  |  |  |  |
| **16** | So099 |  |  |  |  |
| **17** | Aa 245 |  |  |  |  |
| **18** | LMG5031 |  |  |  |  |
| **19** | C17 |  |  |  |  |
| **20** | C19 |  |  |  |  |
| **21** | Lo049 |  |  |  |  |
| **22** | Br642 |  |  |  |  |
| **23** | Li012 |  |  |  |  |
| **24** | Is579 |  |  |  |  |
| **25** | C |  |  |  |  |
| **27** | Lw1047 |  |  |  |  |
| **28** | Br257 |  |  |  |  |
| **29** | Br667 |  |  |  |  |
| **30** | C18 |  |  |  |  |
| **31** | LMG2107 |  |  |  |  |
| **32** | Bu004 |  |  |  |  |
| **33** | C1 |  |  |  |  |
| **34** | PAO29 |  |  |  |  |
| **35** | C13 |  |  |  |  |
| **36** | C2 |  |  |  |  |
| **37** | Aa 246 |  |  |  |  |
| **38** | LMG14084 |  |  |  |  |
| **39** | Br735 |  |  |  |  |
| **40** | Mi162 |  |  |  |  |
| **41** | SG17M |  |  |  |  |
| **42** | Pr335 |  |  |  |  |
| **43** | Is580 |  |  |  |  |
| **44** | Li009 |  |  |  |  |
| **45** | Be136 |  |  |  |  |
| **46** | Is573 |  |  |  |  |
| **47** | Br908 |  |  |  |  |
| **48** | TuD199 |  |  |  |  |
| **49** | So095 |  |  |  |  |
| **50** | So092 |  |  |  |  |
| **51** | Br229 |  |  |  |  |
| **52** | SG50M |  |  |  |  |
| **53** | PT31M |  |  |  |  |
| **54** | Mi151 |  |  |  |  |
| **55** | US447 |  |  |  |  |
| **56** | Bo548 |  |  |  |  |
| **57** | Br680 |  |  |  |  |
| **58** | PA6 |  |  |  |  |
|  | Summary | 39 | 34 | 14 | 27 |
|  | % of the tested strains | 67, 24 | 58,62 | 24,14 | 45,55 |

Grey box –active, white box – no activity.
