# Supplementary material for: Characterization of the Newly Isolated Lytic Bacteriophages KTN6 and KT28 and Their Efficacy against Pseudomonas aeruginosa Biofilm
Source: PLoS One. 2015 May 21;10(5):e0127603. doi: 10.1371/journal.pone.0127603 (PMC4440721; doi:10.1371/journal.pone.0127603)
Supplement: S2 Table — (DOCX) [file pone.0127603.s004.docx]

**S2 Table. Major features of characterized phages.**

| **Phage** | **KT28** | **KTN6** |
| --- | --- | --- |
| **Water source** | Irrigation field | |
| **City/Country/ Date** | Wroclaw/Poland/ 2011 | |
| **Host** | PAO1 | |
| **Genome size (bp)** | 66,381 | 65,994 |
| **GC (%)** | 55,6 | 55,51 |
| **ORFs** | 94 | 92 |
| **Unique ORFs** | 1 | - |
| **Virion dimensions head/tail [nm]** | 74/136 | 72/123 |
| **Latent period [min]** | 35 | 35 |
| **Burst size [pfu/cell]** | 65 | 96 |
| **Heat stability for 1h [ºC]** | 40-70 | 40-70 |
| **pH stability for 1h at RT** | 3-12 | 3-12 |
| **Chloroform sensitivity at RT/4 ºC [h]** | 24 | 24 |
| **Phage infectivity inhibition by LPS (PI_50_) [ug/ml]** | 36.0 | 43.3 |
| **LPS-binding assay [OD 450nm]** | 0.74 | 0.75 |
| **Biofilm eradication [CFU reduction]** | 70-80 % | 70-90 % |
| **Pyocyanin production inhibition in biofilm structure** | + | + |
| **Pyocyanin production inhibition in biofilm structure** | + | + |
| **Increase of diffusion though biofilm structure** | + | + |
| **Biofilm eradication measured by goniometry analysis** | + | + |
