# Supplementary material for: Characterization of the Newly Isolated Lytic Bacteriophages KTN6 and KT28 and Their Efficacy against Pseudomonas aeruginosa Biofilm
Source: PLoS One. 2015 May 21;10(5):e0127603. doi: 10.1371/journal.pone.0127603 (PMC4440721; doi:10.1371/journal.pone.0127603)
Supplement: S3 Table — Percent Identity Matrix—created by Clustal2.1 (http://www.ebi.ac.uk/Tools/msa/clustalw2/). (DOCX) [file pone.0127603.s005.docx]

**S3 Table. The comparison of KT28 and KTN6 to 7 other Pb1-like phages (14-1, BcepF1, F8, LBL3, LMA2, PB1 and SN) based on nucleotide similarity.**

| **Phage** | **14-1** | **BcepF1** | **F8** | **LBL3** | **LMA2** | **PB1** | **SN** | **KT28** | **KTN6** |
| --- | --- | --- | --- | --- | --- | --- | --- | --- | --- |
| **14-1** | 100.00 | 46.47 | 89.41 | 92.88 | 93.67 | 89.23 | 95.31 | 94.46 | 93.37 |
| **BcepF1** | 46.47 | 100.00 | 44.97 | 45.90 | 46.88 | 44.77 | 46.68 | 47.74 | 46.94 |
| **F8** | 89.41 | 44.97 | 100.00 | 91.06 | 89.00 | 94.49 | 89.01 | 89.33 | 89.86 |
| **LBL3** | 92.88 | 45.90 | 91.06 | 100.00 | 92.24 | 91.23 | 92.69 | 92.47 | 92.35 |
| **LMA2** | 93.67 | 46.88 | 89.00 | 92.24 | 100.00 | 87.61 | 94.53 | 96.19 | 96.55 |
| **PB1** | 89.23 | 44.77 | 94.49 | 91.23 | 87.61 | 100.00 | 87.53 | 88.35 | 88.54 |
| **SN** | 95.31 | 46.68 | 89.01 | 92.69 | 94.53 | 87.53 | 100.00 | 95.64 | 94.52 |
| **KT28** | 94.46 | 47.74 | 89.33 | 92.47 | 96.19 | 88.35 | 95.64 | 100.00 | 96.43 |
| **KTN6** | 93.37 | 46.94 | 89.86 | 92.35 | 96.55 | 88.54 | 94.52 | 96.43 | 100.00 |

Percent Identity Matrix – created by Clustal2.1 (http://www.ebi.ac.uk/Tools/msa/clustalw2/).
