# Supplementary material for: Characterization of the Newly Isolated Lytic Bacteriophages KTN6 and KT28 and Their Efficacy against Pseudomonas aeruginosa Biofilm
Source: PLoS One. 2015 May 21;10(5):e0127603. doi: 10.1371/journal.pone.0127603 (PMC4440721; doi:10.1371/journal.pone.0127603)
Supplement: S4 Table — (DOCX) [file pone.0127603.s006.docx]

**S4 Table. Clustering coefficient and betweenness centrality in the protein-sharing network of phage Aaphi23, and Pb1-like/Bcep781-like phages.**

| **Names** | **Clustering coefficient** | **Betweenness centrality** |
| --- | --- | --- |
| **Aaphi23** | 0,56084656 | 0,10306764 |
| **JG024** | 0,76679842 | 0,03102882 |
| **141** | 0,91904762 | 0,0092124 |
| **PB1** | 0,91904762 | 0,0092124 |
| **KT28** | 1 | 0 |
| **KTN6** | 1 | 0 |
| **SN** | 1 | 0 |
| **BcepF1** | 1 | 0 |
| **OP2** | 1 | 0 |
| **Bcep781** | 1 | 0 |
| **KPP12** | 1 | 0 |
| **LBL3** | 1 | 0 |
| **BcepNY3** | 1 | 0 |
| **LMA2** | 1 | 0 |
| **Bcep1** | 1 | 0 |
| **Bcep43** | 1 | 0 |
| **F8** | 1 | 0 |
| **ECML** | 1 | 0 |
| **SPM1** | 1 | 0 |
| **NH-4** | 1 | 0 |
| **BcepB1A** | 1 | 0 |
