# Supplementary material for: Characterization of the Newly Isolated Lytic Bacteriophages KTN6 and KT28 and Their Efficacy against Pseudomonas aeruginosa Biofilm
Source: PLoS One. 2015 May 21;10(5):e0127603. doi: 10.1371/journal.pone.0127603 (PMC4440721; doi:10.1371/journal.pone.0127603)
Supplement: S1 Text — (DOCX) [file pone.0127603.s007.docx]

## **Cultivation methods**

PET membranes with biofilm of formed 24, 48 and 72 h at 37°C in TSB medium after incubation with phages were stained by CV (0.004%) for 15 min or were tested on the level of pyocyanin and pyoverdin in supernatants. The membrane probes stained by CV were incubated with 30% acetate acid to release crystal violet from biofilm and the level of the stain was determined at 531 nm by a Microplate Reader TECAN Infinite 200 PRO (**Tecan Group Ltd.,** Switzerland) [26]. The absorbance of pyocyanin in medium (200 µl of bacterial cell free supernatant) was determined spectrophotometrically at 691 nm [27]. The fluorescence of pyoverdin was analyzed (λ_ex_ = 398 nm; λ_em_ = 460 nm) in supernatants with a TECAN Infinite 200 PRO microplate reader (**Tecan Group Ltd.,** Switzerland) [28]. All experiments were performed in triplicate.

## **Laser interferometry**

The degradation of biofilm is associated with increase of the permeability of its matrix for low molecular mass compounds. The quantitative measurements of cultivation medium (TSB) diffusion through biofilm structure after incubation with phages might indicate disruption of its structure. This lytic activity of phages against PAO1 biofilm was tested by a laser interferometry method. At the first step of analysis, the percentage level of membrane covered by biofilm was estimated. The images of membrane covered by PAO1 biofilm were stained by CV, converted to grey-scale digital images and analysed with ImageJ computer imaging software program [29]. The value 1 denotes black colour and value 256 denotes white colour. A native membrane was used as control. The measurement set-up for the interferometric investigations of the substance transport was presented previously [30–33]. The measurement set-up consisted of a Mach-Zehnder interferometer with an He-Ne laser, a system of two measurement cuvettes, a TV-CCD camera, and a computer with a system for the acquisition and processing of interference images. The system under study consists of two glass cuvettes (internal dimensions: 70 mm high, 10 mm wide, 7 mm optical path length) separated by the horizontally located membrane (PET membrane with PAO1 biofilm formed for 72 h at 37ºC and incubated with phages). The lower cuvette was filled with an aqueous 3% TSB solution while in the upper cuvette contained pure water. With such a configuration of the measurement system, the solution in the upper cuvette remains undisturbed and a stable concentration boundary layer (CBL) of thickness *δ* is created. The TSB concentration is uniform in the planes parallel to the biofilm-solution interface and concentration gradients occur only in the vertical direction. The interferograms, which appear due to the interference of two laser beams, are determined by the refraction coefficient of the solute, which in turn depends on the concentration of the TSB medium. Recording the interferograms with a given time-step (Δ*t*=2 min*.*) one can reconstruct (after computer analysis of interference fringe course in these interferograms) the spatio-temporal concentration distribution i.e. concentration profile *C(x,t).* The amount of TSB medium, *N*(*t*), which diffuses in time *t* through biofilm formed on PET membrane to water was calculated by integrating the concentration profile *C(x,t)* in upper cuvette according to:

$$N(t)=S\int_{0}^{\delta} C(x,t)dx$$

where *S* denotes the surface of the biofilm-water interface (*S*=7×10^-5^ m^2^), and *δ* the CBL thickness (determined interferometrically). All experiments were performed at a temperature of 37°C.

## **Goniometry**

The hydrophobicity of PET membrane was tested by measuring the contact angle using the static sessile drop method. The values of contact angle were determined for TSB medium drop on PAO1 biofilm surface formed for 72 h on PET membrane, before and after incubation with KT28 or KTN6 phage. Native PVDF and PET membranes were used as control. The results are presented as the means ± SD from three independent experiments. The goniometer OCA 15Ec (Dataphysics, Germany) was used to determine the value of the contact angle which is positively correlated with the hydrophobicity of the tested surface.

## **Data analysis**

The data were analyzed using the Statistica software package (StatSoft, Tulsa, OK, USA). Analyses were done in triplicate for cultivation methods and in three independent experiments for laser interferometry and goniometry analyses. All the values in this study are expressed as mean ± SD. If no significant differences between variations were found by means of the Snedecor-Fisher test, the differences were compared using one-way ANOVA.
